# How do parents scaffold their autistic children’s bilingual language interactions in everyday settings?

**DOI:** 10.1177/13623613251355259

**Published:** 2025-07-27

**Authors:** Shana R. Cohen, Alison Wishard Guerra, María José Aragón, Angeline Estrada, Eunsu Lee

**Affiliations:** 1 University of California San Diego, USA; 2 University of California, Los Angeles, USA

**Keywords:** activity settings, autism spectrum disorders, bilingual autistic children, communication and language, decontextualized language, environmental factors, multilingual autistic children

## Abstract

This study examines how multilingual caregivers use their linguistic resources to support their autistic children’s language development, challenging historical narratives recommending therapists use only one language, English. Using a critical language socialization framework in which multilingualism is considered a practice that transcends linguistic competence, we analyzed 108 videos from five Spanish-dominant, Mexican heritage families. Findings showed that (1) families flexibly used their linguistic resources in daily interactions; (2) home activity settings predicted language use in distinct ways, transportation activities provided fewer constraints for interlocutors to use decontextualized language, language for abstract reasoning and prediction; and (3) variability in maternal scaffolding strategies influenced children’s decontextualized language. We recommend the implementation of asset-based interventions designed to leverage the strengths of multilingual autistic children and their families.

**Lay Abstract:**

Mexican heritage bilingual mothers of autistic children use a variety of language practices when talking with their children. We asked mothers to video record their language interactions with their autistic child over 10 days. Specifically, we analyzed the verbal language practices parents and children used during daily routine activities (e.g. meals). Historically, therapists have recommended that multilingual families only use English when talking with their autistic children, so as not to confuse them and interfere with their development. It is important to understand how multilingual parents use this non-empirical advice. In our sample, we found that mothers used a variety of language strategies including Spanish, English, describing and labeling their environment, action directions (directing a child to perform an action), close-ended questions (e.g. what color is this?), open-ended questions (e.g. How does the radio work?), and abstract reasoning to interact with their children. These strategies varied across activity settings. In particular, transportation was an important setting for children to use abstract language. Mothers’ verbal strategies influenced children’s language output, including children’s use of abstract reasoning and prediction, one of the most complex language strategies.

Caregivers’ multilingual interactions with young children are a powerful socializing mechanism, reflecting broader cultural beliefs about learning, the roles of children and adults, and the use of multiple language systems across activity settings (Garrett & Baquedano-López, 2002; Schieffelin & Ochs, 1986). Multilingual children who use their home language can develop proficiency across academic disciplines (e.g. reading), in both their home language and in English (Bialystok & Craik, 2010; Wagley et al., 2022). Few studies have examined how multilingual caregivers utilize their linguistic and cultural resources across activity settings to enhance their autistic child’s language and development (Cohen et al., 2023; Gilhuber et al., 2023).

For families rearing multilingual autistic children, common misperceptions about bilingualism (e.g. “English only is best”) persist among therapists and educators (Peña, 2016). As a result, therapists have recommended pedagogical approaches and interventions that discourage families from using their native language during therapy and daily routine activities (Levey & Sola, 2013; Nayeb et al., 2015; Peña, 2016). Imagine a scenario in which families are prevented from using their native language. The child may feel isolated; they may miss opportunities to test out vocabulary, learn from caregivers or siblings who may serve as home language models, and develop strong relationships (Tseng & Fuligni, 2000). Family members may also feel disconnected (Kremer-Sadlik, 2005). Studies show that multilingual families feel more comfortable interacting with their autistic child in their native language (Franco & Costa, 2024).

Limited research has identified—and capitalized upon—multilingual families’ interactional resources and the culturally situated environments that promote language learning (Cohen et al., 2023; Gilhuber et al., 2023; Yu, 2016). Multilingualism, a practice that transcends linguistic competence, includes activities and interactions that vary according to interlocutors’ environmental conditions (Baquedano-López & Garrett, 2023). In Yu’s (2016) case study, the autistic child used code-switching as a tool to interact with family members who spoke Mandarin and English. More recent studies show selective cognitive benefits (once considered signature deficits of an autism diagnosis) for bilingual autistic children. Benefits include improved cognitive flexibility and executive functioning, the ability to understand “false beliefs,” and more language production as compared to their monolingual autistic peers (Ge et al., 2024; Gonzalez-Barrero & Nadig, 2017; Peristeri et al., 2021a, 2021b, 2024). These promising findings can potentially transform intervention efficacy for multilingual autistic children.

## Parent–child interactions at home predict language outcomes

Considerable research has demonstrated how parenting practices (e.g. parental responsiveness and their complex linguistic input) can optimize home language environments for monolingual, neurotypical children (Anderson et al., 2021; Landry et al., 2006; Smith et al., 2024). Anderson and colleagues (2021) found that the parental language quality predicted child language outcomes more than parental language quantity, and these findings did not vary by socioeconomic status or child gender. In a study using acoustic analysis of turn-taking in mother–child dialogue, Smith et al. (2024) found that mothers with more consistently timed language responses had children with more vocabulary. The context of the learning environment supports child development beyond the complexity of the language input itself (Cox et al., 2023).

Home settings offer multiple language learning opportunities that align with families’ culturally informed childrearing beliefs and inform how parents enact language elicitation strategies (e.g. Bridges et al., 2012, 2022; Correa-Chávez & Rogoff, 2009). For example, some immigrant Mexican families who rear both typically and atypically developing children, may prioritize the notion of “bien educado”—the expectation for children to show respect for their elders by not speaking unless a direct question is asked of them (Bridges et al., 2012, 2015; Cauce & Domenech-Rodríguez, 2002). Other studies have shown variations in how culturally diverse families organize children’s learning, ranging from a model of direct instruction favored in Western schooling to a model of collaboration and intent observation found among some indigenous communities across the Americas (Correa-Chávez & Rogoff, 2009; Rogoff et al., 2015). For example, Correa-Chávez and Rogoff (2009) found that children from a Guatemalan Mayan heritage community demonstrated more sustained attention and had more advanced learning outcomes during an opportunity to learn through observation than did children from a European American heritage community. These studies demonstrate that home settings are informed by cultural child rearing values that drive how parents organize learning opportunities in everyday routines, highlighting distinct strengths within and across cultural groups.

For autistic children, home environments offer multiple opportunities for interlocutors to test out new vocabulary and syntax. These repeated, daily routine interactions are important contexts for autistic children who may need repeated opportunities to practice language (e.g. requesting a favorite snack, or sharing a toy with a sibling). Research has shown that as compared to non-autistic children, autistic children need more strategic, routine support in developing their language and communication skills (Tager-Flusberg & Kasari, 2013; Tubul-Lavy et al., 2020). The home has long been considered a consequential setting for autistic children as it provides flexible, culturally situated, individualized, and multiple language learning opportunities (Cohen et al., 2023; Hampton & Kaiser, 2016; O’Hagan et al., 2021).

## Language scaffolding strategies

Children’s language development depends on both the quality and quantity of linguistic input (Paradis et al., 2011). Fusaroli et al. (2019) studied 32 autistic preschoolers and their typically developing peers; parents with longer mean length of utterances (MLUs) led to children producing more words and longer MLUs over time. Similarly, Swanson et al. (2019) found that infants exposed to more words and conversational turns at home had better receptive and expressive language skills by age two. Studies have also explored how parents use language scaffolding strategies, such as open/close-ended questions, elaborations, repetition, and contextualized/decontextualized language (Tamis-LeMonda et al., 2012). Dieterich et al. (2006) examined maternal language scaffolding (e.g. questions, directives) in 269 low-income mothers with term and preterm infants over 10 years. At ages 3 and 4, rich language scaffolding during natural play and toy activities promoted children’s language skills and later reading comprehension. Similarly, Jiang et al. (2024) found that parents’ echoing style—repeating children’s language—was linked to better child executive function outcomes, even after accounting for child language.

Similar associations—parent language scaffolding associated with children’s language output—have been found in autistic populations (McDuffie & Yoder, 2010; Naigles, 2013). Smith et al. (2023) studied 53 parent–child dyads during 10 minutes of free, open-ended playtime, and found that parents’ language imitation and expansion predicted autistic preschoolers’ language several months later. Pierucci (2016) studied mothers’ scaffolding strategies of three autistic toddlers during play time. An increase in parent scaffolding during play (e.g. comments, requests, prompts) led to child engagement in pretend play. Parents’ language scaffolding provides children with guided questions and comments that support autistic children’s language production.

One key measure of language quality that predicts later academic, cognitive, and language outcomes is decontextualized language—talk about non-present events or abstract concepts (Conica et al., 2023; Rowe, 2013). These interactions often involve narratives or stories about past/future events or play acting, using complex sentences, and low-frequency vocabulary. In contrast, contextualized language typically includes high-frequency words and visual cues from the present environment. Studies show that decontextualized language enhances children’s vocabulary and syntax (Demir et al., 2015), predicts academic language use into adolescence (Uccelli et al., 2019), and improves numeracy and literacy skills in middle childhood (Conica et al., 2023).

Few studies have explored how parents scaffold decontextualized language with autistic children (Pierucci, 2016). Research shows variability in narrative development among mono- and bilingual autistic children, with some narratives reflecting syntactic complexity akin to decontextualized language (Peristeri et al., 2017, 2020). Other studies have examined decontextualized scaffolding in atypical populations, such as children with language impairments (Gillam et al., 2012) or Down syndrome (Hilvert et al., 2021). However, there is limited research on parent scaffolding in multilingual autistic children (Bottema-Beutel et al., 2019; Oudet et al., 2024), despite multilingual families having language resources (i.e. multilingual interactions) for supporting decontextualized language. Future studies should capitalize on these resources to develop interventions that incorporate more complex scaffolding strategies, informed by culturally situated practices and home contexts, to better support children’s expressive language development.

## Theoretical framework

This study is guided by sociocultural theory (Rogoff, 1990) and critical language socialization theory (Baquedano-López & Garrett, 2023; Garrett & Baquedano-López, 2002; Ochs & Schieffelin, 2011). Sociocultural theory views children’s learning and development as situated within everyday family activities, shaped by novice-expert relationships. Learning is a culturally and historically mediated process, varying across communities, where children acquire skills and practices meaningful to their families and communities (Gutiérrez & Rogoff, 2003; Rogoff et al., 2015). Language socialization theory focuses on “socialization through language and socialization to use language” (Schieffelin & Ochs, 1986), considering language as a tool for transmitting cultural knowledge and norms through social interaction. Routine activities, especially those involving complex caregiving, are key sites for observing how families use language. Language socialization theory guides researchers to utilize an emic approach, examining within group language practices, to understand how members within a linguistic and cultural group understand and use their culturally situated language practices (Baquedano-López & Garrett, 2023; Garrett & Baquedano-López, 2002).

We adopt a dynamic, socioculturally grounded view of children’s identities as bilinguals and multilinguals (e.g. García, 2009; García & Wei, 2014). In this article, we use the term “multilingual” to highlight children’s potential in developing skills across multiple languages and language varieties over time rather than more deficit-oriented terms, such as “English Learner,” often used in education settings. This perspective views multilingual children’s identities as deeply intertwined with their linguistic practices: “Language use is tied to the representation of self and the social world and how positive representations of identities are tied to language use” (Baquedano-López & Garrett, 2023, p. 53). We focus on understanding how bilingual families’ language practices develop in their home environment and support children’s language use. This perspective allows multilingual children to be recognized for the full range of their linguistic knowledge and skills.

## The current study

For multilingual families raising neurodivergent children, there is limited research on daily language interactions in context. Recent calls from developmental researchers have emphasized the need to replace deficit models with culturally situated models that explore within group heterogeneity and identify adaptive cultural practices that promote positive outcomes, including linguistic strengths and social competence (Cabrera, 2013), to examine the “multiple layers of social context” that influence language learning for autistic children (Bottema-Beutel & Kim 2021, 2024; Smith et al., 2023). These scholars advocate for studies that explore how the language environment relates to autistic children’s language development, incorporating both automated language analysis and human coding to understand how the complexity and syntactic diversity of caregiver talk affects child language and interactions. Smith et al. (2023) and others also urge investigations into specific parent language scaffolding strategies that promote child language acquisition. Our study responds to these calls by focusing on language scaffolding strategies within multilingual families. We address these gaps with three research questions:

1. What language practices do mothers and their autistic children use during daily routine interactions?
2. Does the activity setting predict mothers’ language practices, after controlling for maternal education?
3. Do specific types of maternal language practices significantly predict child language production (e.g. average number of words spoken per minute), after controlling for maternal education?

## Methods

### Participants

The families in this study were participants in a larger investigation examining autism spectrum disorder (ASD) beliefs and diagnostic pathways among 38 Mexican heritage, Spanish-speaking families raising at least one autistic child (Cohen et al., 2018, 2020, 2023). Families were recruited from a medical clinic near the U.S.–Mexico border and a non-profit California Department of Developmental Services Regional Center. Recruitment packets were sent to families with autistic children, and interested parents were screened for eligibility (e.g. availability, interest, autism diagnosis, Hispanic heritage, Spanish-speaking parent). Five mother–child bilingual (Spanish-English) dyads participated in this sub-study. Mothers were born in Mexico, immigrated to Southern California between the ages of 12 and 29, and spoke Spanish as their first language. Children’s exposure to English in the home varied: three families reported speaking “some” English (e.g. the child spoke English to siblings), one family used Spanish with their grandparents and English with their parents, and one family primarily spoke English with some Spanish. Household income ranged from under $15,000 to $35,000 USD, with an average household size of 5.2 people (range: 2–10). The mean age of the target children was 5.2 (range: 4–6) years, and all target children were male. See Tables 1 and 2 for additional participant demographic characteristics.

**Table 1.** Mother demographic data.

Family pseudonym	Age	Highest degree obtained	Years in US (age of immigration)	Occupation	Primary home language	Household size (annual income, USD)	Cultural or linguistic practices
Huerta	41	Elementary school	27 years (14 years)	Waitress at fast-food chain	Spanish (household) English (child)	2 ($15,001–$25,000)	Bilingual (English-Spanish) home language usage; Mother understands “70%–80%” of English (self-reported)
Fernandez	30	Bachelor’s (in progress)	2 years (28 years)	Homemaker	Spanish	4 ($25,001–$35,000)	Family lives along U.S.—Mexico border, frequently visits relatives in Mexico
Benitez	34	High school	5 years (29 years)	Not employed	Spanish	4 ($15,001–$25,000)	English is the family’s secondary language
Herrera	48	Middle school	36 years (12 years)	Swap meet merchant	Spanish	10 (Under $15,000)	English is the family’s secondary language; Mother does not know English (self- reported; “yo no sé hablar inglés, entonces ahí nos complicamos un poquito”^a^)
Robles family	28	High school	15 years (13 years)	Not employed	Spanish, but both parents prefer to speak English to target child	6 ($15,001–$25,000)	Family uses Spanish at home. They use English to speak to the child “as to not confuse” the child. School and therapy sessions are in English. Both paternal grandparents live with family and speak in Spanish to target child

a English translation: “I don’t know how to speak English, so that’s where things get a little complicated.”

**Table 2.** Target child demographic data.

Family pseudonym	Age (sex)	Age of autism diagnosis	Developmental milestones	School context	Past and current therapies (Hours Per Week)	Language practices (Reported by mother)
Huerta	6 (M)	2 years, 3 months (by pediatrician) 3 years (by school district)	Speech and motor delays at 2 years	Attends English-speaking general education classroom with special education services	Speech (10); Occupational (20) In-Home Therapy (hours not specified); Speech and Psychological Services at school (2)	Child reported to distinguish when to speak in either Spanish or English (e.g. independently speaks in Spanish when calling relatives in Mexico); Home therapists were bilingual and provided therapy in English, spoke to Mother in Spanish; Child reported to speak more English than Spanish in home
Fernandez	4 (M)	3 years (by regional center)	Mother reported typical developmental milestones until 18 months	General education classroom 5 days/week; School language not specified	Unspecified therapy at two centers in Mexico (5); In-home Applied Behavioral Analysis (10)	Spanish (primary in-home), English (secondary in-home)
Benitez	5 (M)	3 years, 1 month (by regional center)	Speech delays at 3 years	General education classroom; School language not specified	Applied Behavioral Analysis (hours not specified); Speech at school (0.5);	Spanish (primary in-home), English (secondary in-home)
Herrera	6 (M)	2 ½ years (by regional center)	Speech delays at 2 years	General education classroom; School language not specified	Cognitive therapy (5 days/week; hours not specified)	Spanish (primary in-home), English (secondary in-home)
Robles	5 (M)	Approximately 3 years (by regional center)	Vocabulary delay at 2 years	Special education class at a regular English-speaking school	Applied Behavioral Analysis (10); Group speech at school (0.5)	Mother prefers to use English “so as to not confuse target child” as his school and therapy sessions are in English; Grandparents (who live in the same household) speak in Spanish to target child

### Data collection

To examine mother–child daily language interactions, families video-recorded their interactions with their autistic child during routine daily activities for at least 20 minutes over 10 consecutive days. This methodology is similar to other studies using discourse analysis (Sterponi & de Kirby, 2016). Participants completed Institutional Review Board (IRB)-approved consent forms and received a tripod and camera training with operating instructions for the digital video recorders. Families were asked to engage naturally, across contexts (e.g. home/school), while recording daily, routine activities and interactions in which they were interacting with their autistic child. During camera training, parents and researchers brainstormed examples of daily routine activities in which families could video-record (e.g. child and parent cooking/eating together). Ultimately, parents decided which family members were included, when, and for how long they would record. Families were informed that they could halt filming and/or withdraw consent for the videos and associated data at any time. After 10 days, a researcher collected the video recorders, retrieved the footage, and invited each mother to participate in a brief audio-recorded “post” interview to share their experiences with the data collection. All mothers expressed confidence that the recordings captured their families’ naturally occurring daily activities. A total of 179 videos were recorded, with families recording between 9 and 44 videos. Videos were excluded if they captured fewer than five conversational turns between the mother and the target child. While other family members’ talk was transcribed, it was not included in this analysis. The final dataset comprised 108 videos, ranging from 10 seconds to 39 minutes in length.

### Community involvement statement

The study participants included mothers of autistic children who were co-designers of the study as they determined the contexts and quantity of video recordings collected throughout their daily, routine activities. The research questions were informed by the current gaps in the literature in which multilingual families are encouraged to speak to their children in English only. Findings will be used to support families’ use of their home language during interventions.

### Data analysis

Video recordings of interactions were transcribed verbatim using the Codes for the Human Analysis of Transcripts (CHAT) transcription system, a standardized format for analyzing naturalistic language samples (MacWhinney, 2000). Transcriptions were conducted by trained coders and verified for accuracy by a second researcher to ensure reliability. An utterance was defined as a word, phrase, or independent clause (including a verb) (Bernstein Ratner & Brundage, 2024; Kanaya & Santiago, 2019; MacWhinney, 2000). Vocalizations separated by a 2 second (or longer) pause were counted as two distinct utterances, even if neither constituted an independent clause (MacWhinney, 2000; Bernstein Ratner & Brundage, 2024). Monosyllabic utterances, such as “Mhm,” were labeled as “(affirmative)” or “(negative),” and unintelligible utterances were marked “[UI]” or “[SL]” if uncertain. Pauses shorter than 10 seconds were marked with bracketed ellipses. Each transcript was cross-checked with its corresponding video by a second coder to ensure accuracy in both English and Spanish.

To identify the activity settings during language interactions, transcripts were reviewed alongside the video recordings and coded based on the family’s main activity (>50% of the recording time). The majority of transcripts (84%) included only one activity setting. For all transcripts that included more than one activity setting (*n* = 17), the length of time for each activity setting was individually assessed to determine the majority activity. All of the transcripts included one of the following activity settings for more than 50% of the recording time. Activity settings were categorized as follows: Daily Living (e.g. personal hygiene, chores, TV, bedtime routines), Reading/Homework (e.g. reading, homework assistance), Family Mealtime (e.g. preparing or eating food), Play (e.g. games, sports, play with toys), and Transportation (e.g. walking or traveling by car).

For a contextually sensitive analysis of language scaffolding strategies, each utterance was coded by communicative function type (Tamis-LeMonda et al., 2012), with four primary categories for parents’ language: scaffolding, contextualized reference, decontextualized reference, and questions. All utterances that did not fall under one of the four categories were coded with other codes (e.g. reading/singing, onomatopoeia, not-child directed speech, other) and were not included in the analysis. Utterances coded as “other” ranged in frequency of occurrence .1%–6% of child utterances and from 1%–2% of parent utterances. Parent scaffolding included behaviors like expanding on the child’s utterance, backchanneling (e.g. “uh huh”), repeating, confirming, or prompting the child to repeat. Child language codes included the same categories for communicative practices (i.e. contextualized and decontextualized references; open-ended and closed-ended questions), but did not include “scaffolding” codes since those were defined as strategies employed by parents to support their child’s language use (Please see Supplementary Material for content code definitions and excerpts).

Contextualized references involved describing or labeling the immediate environment. Decontextualized references were defined as language used to refer to things beyond the present moment and context (e.g. “The rainbow in the sky yesterday appeared because it rained.”). Questions were further divided into two subcodes: (1) closed-ended (e.g. “Do you like puppies?”) and (2) open-ended (e.g. “What do you think happened next?”). Some utterances were tagged with multiple subcodes (e.g. questions were double coded with contextualized or decontextualized reference). All utterances were coded for language use (e.g. Spanish only, English only, bilingual). Proportion scores for each variable were calculated in SPSS by aggregating coded utterances by transcript (see Supplementary Appendix B, Table 1 for code definitions, labels, and examples). Each author coded all transcripts within one family. To obtain interrater reliability, approximately 20% of the transcripts were independently coded by a second coder. Coding dictionary refinement and coding discrepancies were resolved during weekly meetings.

To analyze language production, transcripts were processed using CLAN (computerized language analysis), part of the CHILDES suite (MacWhinney, 2000). CLAN was used to compute measures of language production for both mother and child, including number of utterances, mean length of utterance in words (MLUw), type-token ratios (TTR), words per minute (WPM), and word frequency in both Spanish and English.

## Results

### Description of language practices

For research question one, parents and their autistic children used a variety of language practices during daily routine interactions across five activity settings, including Spanish and English, multiple language production strategies, and a variety of communicative function strategies to measure language quality. Paired sample T-tests were used to compare mother versus child and English versus Spanish language practices. We found significant differences in maternal and child frequency of Spanish and English utterances. Across all utterances, Mothers used more Spanish (M = 65.7%) than English (M = 21.7%), *t*(df) 107 = 7.30; p < .001. Mothers produced more Spanish utterances than children, (Mother M = 0.66; Child M = 0.44) *t* (107) = −8.29, p < .001. See, Tables 3–6 for descriptive statistics on language use and language practices across activity settings.

**Table 3.** Mother/child descriptive characteristics of language use by activity setting.

Metric	Daily living	Reading/homework	Family mealtime	Play	Transportation	Total
	M (*SD*)	M (*SD*)	M (*SD*)	M (*SD*)	M (*SD*)	M (*SD*)
Number of transcripts	26 (24%)	27 (25%)	18 (16.7%)	18 (16.7%)	15 (13.9%)	108 (%)
*Maternal language use*						
% Spanish utterances	.72 (.30)	.50 (.39)	.75 (.27)	.61 (.37)	.75 (.19)	.66 (.33)
% English utterances	.18 (.30)	.36 (.35)	.16 (.28)	.29 (.34)	.03 (.07)	.22 (.31)
% Mixed utterances	.03 (.07)	.02 (.02)	.01 (.02)	.02(.03)	.07 (.10)	.03 (.06)
% Perfect cognates	.07 (.08)	.12 (.12)	.08 (.07)	.08 (.09)	.15 (.10)	.10 (.10)
*Child language use*						
% Spanish utterances	.58 (.35)	.34 (.36)	.48 (.31)	.37 (.36)	.46 (.30)	.44 (.35)
% English utterances	.25 (.34)	.55 (.40)	.31 (.34)	.45 (.40)	.27 (.29)	.38 (.37)
% Mixed utterances	.03 (.04)	.03 (.04)	.04 (.05)	.04 (.07)	.10 (.11)	.05 (.07)
% Perfect cognates	.13 (.12)	.08 (.12)	.17 (.13)	.14 (.20)	.17 (.11)	.13 (.14)

Mixed utterances include English and Spanish in one utterance. Perfect cognates include words that are the same in both Spanish and English (e.g. “Ok”).

**Table 4.** Mother/child descriptive characteristics of total language (English + Spanish) outcomes by activity setting.

	Daily living	Reading/homework	Family mealtime	Play	Transportation	Total
Metric	M (*SD*)	M (*SD*)	M (*SD*)	M (*SD*)	M (*SD*)	M (*SD*)
Number of utterances						
Mother Child	38.75 (39.61) 29.36 (31.81)	131.33 (155.07) 122.00 (85.52)	83.56 (84.74) 56.11 (62.38)	116.17 (113.67) 100.06 (93.37)	24.35 (33.60) 22.35 (23.02)	80 (106.89) 67.66 (75.96)
Number of words						
Mother Child	158.89 (177.30) 102.39 (117.58)	544.15 (650.15) 598.41 (491.78)	342.33 (381.73) 229.83 (356.07)	437.22 (479.18) 330.61 (336.30)	101.65 (157.2) 66.35 (59.43)	323.16 (451.10) 280.00 (377.94)
Words per minute						
Mother Child	41.05 (21.25) 24.73 (14.43)	36.91 (27.44) 598.41 (491.78)	31.56 (17.80) 19.01 (16.16)	38.27 (17.53) 26.47 (14.27)	49.18 (24.44) 32.82 (14.22)	39.25 (22.66) 28.89 (18.20)
MLU (mean length utterance)						
Mother Child	3.94 (.94) 2.97 (1.22)	3.91 (.99) 4.37 (1.82)	3.60 (.98) 3.08 (1.33)	3.59 (.91) 2.80 (0.95)	3.93 (1.08) 3.08 (1.10)	3.82 (.97) 3.33 (1.47)
TTR (type-token ratio)						
Mother Child	.65 (.17) 0.64 (0.16)	.48 (.18) 0.46 (0.21)	.55 (.20) 0.68 (0.23)	.50 (.20) 0.55 (0.27)	.68 (.14) 0.74 (0.13)	.57 (.19) 0.60 (0.22)
Decontextualized reference						
Mother Child	0.17 (0.19) 0.24 (0.27)	0.13 (0.15) 0.13 (0.12)	0.17 (0.19) 0.24 (0.20)	0.17 (0.18) 0.13 (0.15)	0.45(0.23) 0.53 (0.26)	0.20 (0.21) 0.24 (0.25)

**Table 5.** RQ2 mixed-effects model findings: impact of activity settings on mothers’ language practices.

Effect	Estimate	*SE*	95% confidence interval
***Language quantity factor*** *ICC* *⩽* .*0001*			
Intercept			
Daily living	0.25	0.26	−0.27 to 0.76
Family mealtime	−0.11	0.28	−0.65 to 0.44
Play	−0.12	0.29	−0.69 to 0.44
Transportation	0.48	0.32	−0.15 to 1.10
Mother HS graduate	−0.13	0.20	−0.55 to 0.30
Mother more than HS graduate	1.52***	0.32	0.90 to 2.14
Random effects			
Within study variance	2.68e	5.18e−07	0 - .
Between study variance	0.81	0.11	0.61–1.06
***Maternal Spanish Lexical Diversity*** *ICC* *=* .*81*			
Intercept			
Daily living	0.29***	0.17	−0.03 to 0.62
Family mealtime	0.40*	0.17	0.06 to 0.74
Play	0.03	0.18	−0.32 to 0.38
Transportation	−0.02	0.20	−0.41 to 0.38
Mother HS graduate	0.10	1.17	−2.20 to 2.39
Mother more than HS graduate	−0.12	0.14	−2.94 to 2.70
Random effects			
Within subjects variance	1.35	1.37	0.19 to 9.87
Between study variance	0.31	0.04	0.24 to 0.41
***Maternal Bilingual Complexity*** *ICC* *=* *0.05*			
Intercept			
Daily living	0.13	0.26	−0.37 to 0.63
Family mealtime	−0.08	0.27	−0.61 to 0.44
Play	0.03	0.28	−.52 to 0.57
Transportation	1.36***	0.31	0.75 to 1.97
Mother HS graduate	0.13	0.29	−.43 to 0.70
Mother more than HS graduate	0.13	0.39	−.62 to 0.89
Random effects			
Within subjects variance	0.04	0.08	0.0004 to 3.43
Between study variance	0.75	0.11	0.57 to 0.99
***Maternal acknowledge/extend*** *ICC* *=* *0.24*			
Intercept			
Daily living	−0.24	0.27	−0.77 to 0.29
Family mealtime	−0.13	0.28	−0.69 to 0.42
Play	−0.52†	0.29	−1.10 to 0.05
Transportation	−.11	0.33	−0.75 to .53
Mother HS graduate	−0.27	0.57	−1.38 to 0.85
Mother more than HS graduate	0.09	0.71	−1.31 to 1.49
Random effects			
Within subjects variance	0.27	0.31	0.03 to 2.68
Between study variance	0.83	0.12	0.63 to 1.09
***Maternal Questions*** *ICC* *=* *0.16*			
Intercept			
Daily living	−0.36	0.25	−0.86 to 0.14
Family mealtime	−0.17	0.27	−0.69 to 0.35
Play	0.00	0.27	−0.53 to 0.54
Transportation	−0.30	0.31	−0.90 to 0.31
Mother HS graduate	0.94*	0.43	0.09 to 1.79
Mother more than HS graduate	0.80	0.55	−0.29 to 1.88
Random effects			
Within subjects variance	0.14	0.18	0.01 to 1.74
Between study variance	0.73	0.10	0.55 to 0.97

*** p < 0.000; *p < 0.05 † = approaching p < .08.

**Table 6.** RQ3 mixed-effects model findings: impact of mothers’ language practices on child language production.

Effect	Estimate	*SE*	95% Confidence Interval
***Child MLU*** *ICC* *=* *0.28*			
Intercept			
Mother language:			
Language quantity	0.00	0.12	−0.24 to 0.24
Spanish lexical diversity	−0.23	0.19	−0.60 to 0.14
Bilingual language complexity	0.33*	0.12	0.09 to 0.57
Acknowledge/extend	0.32*	0.13	−0.07 to 0.57
Questions	−0.13	0.14	−0.40 to 0.13
Mother HS graduate	−1.83*	0.73	−3.27 to −0.39
Mother more than HS graduate	−1.23	0.93	−3.05 to 0.60
Random effects			
Within study variance	0.45	0.61	0.03 to 6.23
Between study variance	1.16	0.17	0.88 to 1.54
***Child TTR*** *ICC* *<* *0.000*			
Intercept			
Mother language:			
Language quantity	−0.01	0.02	−0.04 to 0.03
Spanish lexical diversity	0.13***	0.02	0.10 to 0.17
Bilingual language complexity	0.02	0.02	−0.01 to 0.05
Acknowledge/extend	0.01	0.02	−0.03 to 0.04
Questions	−0.01	0.02	−0.04 to 0.03
Mother HS graduate	0.11*	0.04	0.03 to 0.18
Mother more than HS graduate	−0.06	0.06	−0.17 to 0.05
Random effects			
Within subjects variance	0.00	0.00	0.00 to 0.00
Between study variance	0.02	0.00	0.02 to 0.03
***Child words per minute*** *ICC* *=* *0.31*			
Intercept			
Mother language			
Language quantity	0.90	1.82	−2.67 to 4.47
Spanish lexical diversity	−5.49†	2.89	−11.15 to 0.16
Bilingual language complexity	3.02	1.85	−0.60 to 6.63
Acknowledge/extend	3.84*	1.94	0.04 to 7.64
Questions	0.31	2.04	−3.69 to 4.30
Mother HS graduate	−7.89	11.76	−30.92 to 15.15
Mother more than HS graduate	−9.70	14.85	−38.81 to 19.41
Random effects			
Within subjects variance	118.61	148.21	10.24 to 1373.37
Between study variance	264.13	37.73	199.63 to 349.48
***Child decontextualized reference*** *ICC* *=* *0.03*			
Intercept			
Mother language:			
Language quantity	0.08***	0.02	0.04 to 0.12
Spanish lexical diversity	0.09***	0.03	0.04 to 0.14
Bilingual language complexity	0.14***	0.02	0.10 to 0.18
Acknowledge/extend	0.06**	0.02	0.01 to 0.10
Questions	−0.00	0.02	−0.05 to 0.04
Mother HS graduate	−0.05	0.06	−0.17 to 0.07
Mother more than HS graduate	0.04	0.09	−0.21 to 0.13
Random effects			
Within subjects variance	0.00	0.00	9.5e–07 to 1.37
Between study variance	0.04	0.01	0.03 to 0.05

*** p < 0.000; **p < 0.01 *p < 0.05 † = approaching significance at p < 0.08.

Language production was calculated by the number of Tokens (Words), Utterances, words per minute (WPM), MLUw, and type token ratio (TTR) spoken by the mother and the child overall. When comparing maternal and child quantity of language production, we found that mothers (M = 39.25) had a higher rate of production as measured by WPM than their child (M = 28.88), *t*(df) 3.63; p < .001. Mothers (M = 3.82) had a longer MLUw than their children (M = 3.33), *t* (107) = −2.89, p < .01. Children (M = .60) had significantly more variability (vocabulary diversity) in their language use than their mothers (M = .57), *t* (107) = 1.96, p < .001. Mothers’ Spanish language was more lexically diverse and included significantly longer utterances than their English utterances. Mothers’ TTR in Spanish (M = .63) was significantly higher than in English (M = .46), *t*(107) 3.85; p < .001. Mothers had a higher MLUw in Spanish (M = 3.63) as compared to English (M = 1.79), *t*(107) 8.91; p < .001. Children’s TTR in Spanish (M = .65) was also higher compared to English (M = .35), *t*(107) 2.22; p < .05. There were no differences in child MLU in Spanish (M = 2.62) or English (M = 2.52), *t*(107) .45; p > .05.

### Communicative function types

Maternal communicative function types were coded into scaffolding, contextualized or decontextualized language, and questions. Mothers used all three scaffolding strategies, including repeat and confirm (14%), expansion/extension (3%), and requests for repetition (1%). The majority of maternal utterances were categorized as contextualized references, with 44% being describe/label and 16% action/attention. Decontextualized references made up 20% of mothers’ utterances. Mothers used more closed-ended questions (M = 22%) than open-ended questions (M = 6%) with a significant difference, *t*(df) = 107, 9.48, p < .001. In addition, mothers used more contextualized language (describe/label—M = 44%) than decontextualized language (M = 20%), *t*(df) = 107, 6.55, p < .001. Interestingly, children had significantly more decontextualized references (M = .24) than their mothers (M = .20), *t*(107) = 1.96, p < .001.

### Language practices by activity setting

For research question two, we aimed to determine whether the type of activity setting significantly predicted mothers’ language practices. To streamline the variables describing maternal language practices, we conducted principal components analysis (PCA) with varimax rotation, using the scree plot and eigenvalues greater than 1 (Tabachnick & Fidell, 2019) to identify latent language practices. These practices were informed by our conceptual framework, language quality and quantity, and common parent language scaffolding strategies from prior research (Tamis-LeMonda et al., 2012). We performed two separate PCAs: one to characterize maternal language output and another to characterize maternal scaffolding strategies.

To reduce the variables measuring maternal language output, we included six variables (MLUw, WPM, TTR, proportion of Spanish utterances, proportion of bilingual utterances, and decontextualized reference) in the PCA. Three factors were identified, which together explained 75% of the total variance. The first factor, language quantity, is defined by high loadings in MLUw (.84) and WPM (.83) and a low loading in TTR (−.50). The second factor, Spanish lexical diversity, is defined by high loadings in TTR (.74) and Spanish utterances (.85). The third factor, bilingual complexity, is defined by high loadings in bilingual utterances (.90) and decontextualized reference (.58). To reduce the variables measuring communicative function type, we included the two maternal language scaffolding variables (Expansion/Extension, Confirm/Acknowledgment) and the two maternal questions variables (Close-Ended Questions, and Open-Ended Questions) in the PCA. Two factors were identified, which together explained 60% of the variance. The first factor, acknowledge and extend, is defined by high loadings in expansion/extension (.81) and confirm/acknowledge (.75). The second factor, questions, is defined by high loadings in the use of close-ended (.86) and open-ended questions (.57).

To examine the impact of families’ activity settings on mothers’ language practices, while controlling for maternal education, a two-level linear mixed-effects model (Hox, 2010) was conducted using Family ID as a random intercept, to account for the nesting of repeated observations within families using STATA version 18 (StataCorp, 2023). We conducted four separate mixed-effects models with each of the following five factors differentiating mothers language practices across activity settings: (1) language quantity; (2) Spanish lexical diversity; (3) bilingual language complexity; (4) acknowledge and extend; and (5) questions. Reading/homework was selected as the referent group for the activity setting, given the dominant focus on reading and homework as the primary context for academic language development. Mothers’ education is a dichotomous variable identifying the completion of more than high school education compared to high school or less as the referent group.

#### Maternal language quantity

A low intraclass correlation (ICC > 0.000) indicates that almost none of the variance was attributed by family-level differences. In addition, none of the activity setting categories significantly predicted language quantity. Maternal language quantity did not vary by activity setting or family context.

#### Maternal Spanish lexical diversity

A high ICC (ICC = .81) indicates that the variance in maternal Spanish lexical diversity was attributed to family-level differences. In addition, interactions that occurred during family mealtime included significantly higher maternal Spanish lexical diversity as compared to reading/homework (β = 0.40; St Error = 0.15; confidence interval [CI]: 0.06–0.74; p < .05). While family context played a significant role, mealtime activities uniquely predicted the use of more complex and varied maternal vocabulary in Spanish.

#### Maternal bilingual language complexity

A low ICC (ICC = .05) indicates that only a small proportion of the overall variance in maternal bilingual language complexity was attributed to family-level differences. Interactions that occurred during transportation activities included significantly higher maternal bilingual language complexity compared to reading/homework (β = 1.36; St Error = 0.31; CI: 0.76–1.97; p < .001). Transportation activities significantly predicted mothers’ individual use of bilingual language complexity.

#### Maternal acknowledge/extend

A low ICC (ICC = .16) indicates that a small proportion of the overall variance in maternal language scaffolding strategies of acknowledge and extend were attributed to family-level differences. Interactions that occurred during play activities had marginally fewer maternal acknowledge/extend scaffolding strategies as compared to reading/homework (β = −0.52; St Error = 0.29; CI: −1.10 to −0.05; p = .07). Play activities significantly predicted mothers’ use of the scaffolding strategy: acknowledge/extend.

#### Maternal questions

A moderate ICC (ICC = .24) indicates that a moderate proportion of the overall variance in maternal questions was attributed to family-level differences. None of the activity settings significantly predicted maternal questions. The estimated increase in maternal questions was 0.36 lower in the daily routines activity setting compared to reading/homework. The confidence interval showed that plausible effects are compatible with our data range from −0.86 to 0.14.

### Predicting children’s language production

To address research question three, we first examined bivariate correlations between the five maternal language factors and the four child language production outcome variables (MLUw; TTR; words per minute, and decontextualized reference). The significant correlations across many of the child language outcomes and the maternal language factors provided appropriate evidence to continue with a linear mixed-effects model for each child language outcome. To examine the impact of maternal language practices on child language outcomes, while controlling for maternal education, a two-level linear mixed-effects model (Hox, 2010) was conducted using Family ID as a random intercept, to account for the nesting of repeated observations within families using STATA version 18 (StataCorp, 2023). We conducted four separate mixed-effects models with each of the following child language production outcome variables: (1) Child MLU; (2) Child TTR (lexical diversity); (3) Child Words per Minute; and (4) Child Decontextualized Reference. Mothers’ education was a categorical variable: (1) high school graduate and (2) more than high school graduate. Less than high school was the referent group.

#### Child MLU in words

A low ICC (ICC = .28) indicates that a low proportion of the overall variance in Child MLU was attributed to family-level differences. Bilingual language complexity (β = 0.31; St Error = 0.12; CI: 0.07–0.55; p < .01) and maternal scaffolding practices of acknowledge/extend significantly predicted child MLUw (β = 0.33; St Error = 0.13; CI: 0.08–0.58; p < .01). When mothers integrated English and Spanish within one utterance, extended children’s contributions, and modeled abstract reasoning, children produced longer utterances.

#### Child TTR

A low ICC (ICC = 0.00) indicates that a low proportion of the overall variance in Child TTR was attributed to family-level differences. Maternal Spanish lexical diversity predicted child TTR (β = 0.14; St Error = 0.03; CI: 0.09–0.19; p < .001). Mothers’ use of Spanish combined with their use of varied vocabulary, predicted children’s TTR, or the variability of the child’s language.

#### Child words per minute

A moderate ICC (ICC = .31) indicates that a moderate proportion of the overall variance in Child WPM was attributed to family-level differences. Maternal scaffolding practices of acknowledge/extend predicted child words per minute (β = 3.86; St Error = 1.93; CI: 0.09–7.64; p < .05). Mothers who acknowledged and extended children’s contributions across both Spanish and English had children who spoke more words per minute.

#### Child decontextualized reference

A low ICC (ICC = .03) indicates that a low proportion of the overall variance in Child decontextualized reference was attributed to family-level differences. Maternal language quantity (β = 0.08; St Error = 0.02; CI: 0.04–0.12; p < .001), maternal Spanish lexical diversity (β = 0.09; St Error = 0.02; CI: 0.04–0.13; p < .001, maternal bilingual complexity (β = 0.13; St Error = 0.02; CI: 0.09–0.17; p < .001), and maternal acknowledge/extend (β = 0.06; St Error = 0.02; CI: 0.02–0.11; p < .01) predicted child decontextualized reference. Mothers who drew from their multiple linguistic resources (e.g. Spanish and English), encouraged children’s participation through acknowledgment and extension, and modeled complex language through the use of diverse vocabulary, longer MLUs, and decontextualized references, had children who produced more decontextualized language.

## Discussion

In this study, we investigated the diverse language practices of Mexican heritage, Spanish-dominant families raising autistic children in U.S. border communities. Using a sociocultural lens and critical language socialization theory, we framed bilingualism as a dynamic, collaborative process rather than a static linguistic competence (Baquedano-López & Garrett, 2023). Few studies have examined how multilingual families with autistic children leverage their linguistic and cultural resources in daily interactions (Cohen et al., 2023; Peristeri et al., 2020). Recent calls for a “contextually sensitive analysis” challenge researchers to consider how broader social and cultural contexts influence autism research (Bottema-Beutel, 2024). Our findings reveal that (1) families flexibly use their linguistic resources in everyday interactions, (2) home activity settings shape language interactions in distinct ways, and (3) variability in maternal language scaffolding strategies affects children’s use of decontextualized language—language used for abstract reasoning and prediction.

### Families’ flexible use of multilingual resources

Families skillfully leveraged their linguistic resources to engage in complex language interactions. Research question 1 revealed that mothers produced more complex language and engaged in longer conversations with their children when speaking their home language, compared to when speaking English. As a result of these scaffolding strategies, children used decontextualized language in both English and Spanish across routine home activities. This flexible language use by both mothers and children aligns with research on bilinguals’ dynamic and adaptive language practices (Baquedano-López & Garrett, 2023; García, 2009) and challenges deficit-based views that label bilingualism as “interference.” Instead, research question three highlights that mothers’ linguistic input and scaffolding strategies, including their multilingual resources, were important in supporting children’s use of decontextualized language. Children’s decontextualized language production, an indicator of abstract reasoning (Rowe, 2013) has been shown to promote reading comprehension and academic language in adolescence (Uccelli et al., 2019). Our findings suggest that individual differences in maternal language practices, including mothers’ use of bilingual language, are important predictors of child decontextualized reference, independent of family-level clustering. Furthermore, even though children may have used more English than Spanish during their interactions, mothers consistently used their home language to guide and socialize their children’s language use across all daily activities while also strategically drawing on their English skills to adapt to their children’s communicative needs. Even when their children engaged in the societally dominant language, these mothers engaged in highly cognitively demanding work to receptively understand their children’s English language and responded appropriately in their home language.

### Activity settings moderate language interactions

The mixed-effects models in research question 2 found that transportation activities—when mothers were taking their children to and from school, therapy, or errands—provided rich opportunities for high-quality language interactions, including translanguaging and decontextualized language. This aligns with previous studies showing that everyday, routine activities like food sharing can foster vocabulary growth in Latine children, sometimes even more effectively than traditional literacy activities like book reading (Cohen et al., 2025; Leyva et al., 2024). Our findings highlight the significance of routine home activities as key contexts for language learning, especially in Mexican heritage families. The familiarity of these routines allows participants to engage more naturally in conversations, freeing them from having to focus on the logistical steps of the activity. This, in turn, scaffolds both parents and children to engage in more cognitively complex language interactions (Dickinson & Tabors, 2001; Fuligni et al., 2012; Leyva et al., 2024).

For autistic children, transportation activities may offer a unique advantage in fostering complex language interactions. Unlike activities such as reading or doing homework, where there may be more pressure for social behaviors like consistent eye contact, transportation activities are often perceived as a “lower stakes” social environment. In these settings, there is less emphasis on traditional social expectations, such as maintaining eye contact, which can be challenging for some autistic children (American Psychiatric Association, 2013). As a result, these activities might create a more accessible space for children to engage with their parents in language-rich interactions. Without the social demands typically associated with other activities, autistic children may feel more comfortable responding to parents’ complex language prompts. This finding is consistent with studies showing that transportation activities can provide important opportunities for decontextualized conversations in non-autistic children (Marvin, 1995; Marvin & Cline, 2009).

Transportation activities present a compelling context for language learning because they likely occur multiple times a day, they usually do not require cultural artifacts or materials to facilitate a conversation, and the conversations that occur almost always reference past experiences, or events that are not in the current context (i.e. decontextualized language) (Marvin, 1994). In other daily routine activities, such as homework and reading, the cultural artifacts of books and assignments mediate the interactions within that activity setting. In other findings with a similar sample, we found that the English dominant artifacts of school work and books aligned with parent and child interactions in English (Cohen et al., 2023). During transportation activities, there is a release of constraints on the mediating cultural artifacts (Bodrova & Leong, 2024; Rogoff, 2003). Consequently, the interlocutors may feel they can use their language resources more freely.

### Maternal language variability enhances children’s language outcomes

Mothers who engaged in high-frequency, lexically diverse, and cognitively complex language interactions in Spanish and English had children who produced more complex language. More specifically, the mixed-effects models from research question 3 mothers’ complex language scaffolding strategies predicted children’s language quantity including MLUw and WPM, and quality of language output including the child’s language variability (TTR) and their decontextualized language. Similar findings show that mothers’ linguistic and syntactic diversity promotes children’s vocabulary and language production (Rowe, 2022). Studies of autistic children demonstrate that high-quality language scaffolding strategies promote autistic children’s complex, pretend play (Pierucci, 2016). Activities like pretend play, mealtime, and transportation incorporate a higher frequency of decontextualized language, exposing children to more diverse and complex language known to predict learning (Uccelli et al., 2019).

Our study findings also showed that children used more decontextualized language than their mothers, a surprising finding given previous studies repeatedly showing “uneven” progress in autistic children’s language development; bilingual autistic children have shown lexical strengths in grammar and vocabulary but relative weaknesses in figurative language and narrative, key components of decontextualized language (Su et al., 2018, 2024). Language development can also be context-dependent, with children struggling to generalize skills across situations (Sánchez Pérez et al., 2020). While bilingual parents typically use fewer expansions than monolingual parents (Hudry et al., 2018), our findings highlight the value of extending and acknowledging children’s talk in bilingual interactions, with Spanish playing a key role in scaffolding. Culturally informed interventions can support mothers in using home language strategies like expanding utterances, encouraging narratives, and engaging in activities – such as pretend play, mealtime, and transportation—that promote decontextualized language.

### Limitations

There are several study limitations. First, our sample consisted of only five bilingual families, with no comparison group (e.g. monolingual families), who determined the quantity and contexts of their video recordings, leading to uneven sampling across contexts. While this approach provided compelling naturalistic data, caution is needed when generalizing the findings. To address this, we used a mixed-modeling statistical framework to account for the nested structure of our data. In addition, communication was defined solely through spoken language, excluding non-verbal and embodied communication, which may be particularly important for Latine and minimally verbal autistic children. Recent studies suggest that bilingual autistic children may have an advantage in interpreting non-verbal cues (e.g. pointing, eye contact) compared to their monolingual peers (Valicenti-McDermott et al., 2013). We also followed a conservative rule in defining an utterance, which may have led to truncation in some cases. Child MLUw might be underestimated due to retracing initial utterances before completing a longer response. For example, one target child’s verbalization, “yeah, you just gotta take you just got to count to one hundred,” was considered one utterance for the purposes of this article, whereas less-conservative definitions may identify up to three utterances. Future research should consider incorporating retracing into MLU calculations (see Johansson, 2008 for a discussion on MLU measurement). Some studies have used more relaxed standards for defining an utterance or calculating MLU.

### Implications for research, practice, and policy

More research is needed that employs a “contextually sensitive analysis” to identify specific factors that can enhance language outcomes for young children (Bottema-Beutel, 2024). Future studies should include multimodal analyses that account for gestures (e.g. pointing) and body positioning to capture joint attention. This social-pragmatic communication can reveal not only the capacity of multilingual learners but also their communicative intent. Research has shown that autistic children use non-verbal gestures with communicative intent, and such gestures may serve as compensatory strategies for minimally verbal children (Valle et al., 2021). These findings can inform the development of individualized interventions that support communication and language learning for autistic children.

Future research should also incorporate a qualitative analysis of children’s vocabulary and language output to understand the types of words that children are producing. Nouns and verbs can be considered less cognitively complex as compared to mental-state terms like feelings. Recent studies provide a mixed picture related to how monolingual and bilingual autistic children utilize mental state words. Losh and Capps (2003) found that monolingual autistic children used more mental state terms as compared to non-verbal autistic children in their narratives whereas Hoang et al. (2018) found no significant differences in the frequency of mental state word use between bilingual and monolingual autistic children (Hoang, et al., 2018). More research is needed to understand the types of words multilingual children use.

Capacity-building interventions that promote the use of the families’ home language are essential for supporting bilingual autistic children and their families. Practitioners and educators must actively listen and learn to identify specific home language practices that naturally foster language use within routine activities. Our study found that transportation was a key context for supporting complex language development. In many early childhood classrooms, transportation is also a common curricular unit. Educators often share pictures and labels of vehicles (e.g. fire truck, taxi cab) and discuss how they serve the community. Teachers could extend this unit by encouraging families to share their own stories about how they use vehicles or other modes of transportation (e.g. “Who do you drive with? Where do you go? Why?”). These strategies have been successful in eliciting complex language from children and families (Figueras-Daniel & Vazquez, 2024).

Similar to the strategies mothers used during transportation activities, teachers can apply these techniques during classroom transitions. Studies show that short transitions can be meaningful contexts for eliciting decontextualized language. For example, during a walk back to class after a bathroom break, a teacher might ask, “What animals do you see in the clouds?” Regularly posing such questions gives children more opportunities to practice language (Ritchie & Gutman, 2013). Practitioners and therapists can also encourage caregivers to use transportation time for language elicitation, such as asking children to retell a story, or describe an experience during a drive to/from school (e.g. “Tell me about the new robot in your science class”).

Study findings also suggest a need to reconsider language assessment policies in therapeutic and school settings. Current assessments often focus on individual skills rather than the interactive dynamics of conversations. From this study, we learned that autistic children possess a sophisticated multilingual repertoire that traditional assessments may not fully capture. Speech and language pathologists should consider using methods like conversation analysis to better assess the language capacities of autistic multilingual children (Yu & Sterponi, 2023). In doing so, they can document their complex language practices as well as the crucial ways in which their caregivers support their language development across social contexts.

## Supplemental Material

sj-docx-1-aut-10.1177_13623613251355259 – Supplemental material for How do parents scaffold their autistic children’s bilingual language interactions in everyday settings? Supplemental material, sj-docx-1-aut-10.1177_13623613251355259 for How do parents scaffold their autistic children’s bilingual language interactions in everyday settings? by Shana R. Cohen, Alison Wishard Guerra, María José Aragón, Angeline Estrada and Eunsu Lee in Autism
