# Supplementary material for: How do parents scaffold their autistic children’s bilingual language interactions in everyday settings?
Source: Autism. 2025 Jul 27;29(12):3014–31. doi: 10.1177/13623613251355259 (PMC12618715; doi:10.1177/13623613251355259)
Supplement: sj-docx-1-aut-10.1177_13623613251355259 – Supplemental material for How do parents scaffold their autistic children’s bilingual language interactions in everyday settings? [file sj-docx-1-aut-10.1177_13623613251355259.docx]

**Appendix**

**Communicative Function Codes**

The following table names, defines, and provides an example of the codes measuring the types of communicative functions that were used to code each utterance across families and activity settings.

**Communicative Functions Coding Table**

*Parent/Child Language Codes, Definitions, and Examples*

| Name of Code | Definition | Example |
| --- | --- | --- |
| Repetition/Confirms/ Acknowledgement/ Backchanneling | Speaker repeats, confirms, or acknowledges the interlocutor's statement. Backchanneling (e.g., “mhm”) is also a way to engage the listener. | C: "That's milk."  P: "You're right. It's milk." |
| Expansion/Extension/ Elaboration | Parent adds details to expand upon or extend the child's original statement. The parent adds new content or meaning to the child's comment. The parent may also elaborate or clarify their own language. [Parent only code] | C: "¡No más quedan dos semanas!"  P: "Ya falta poquito" |
| Contextualized: Describe, Label | The speaker labels or provides additional information about items or actions that are the family’s present physical environment: a facial expression, feeling, sound, a physical movement, or noun. The parent gives the child contextual cues about their immediate environment. | P: “You look sad.”  C: “This is a red truck.” |
| Action/Attention Directives | The parent provides directives in an effort to change or correct the child’s behavior: the parent may encourage the child to complete a task or discipline the child. Child may utilize this code to gain the parent’s attention. | P: “Andale, finish your homework.”  C: “Mami, look at me!” |
| Decontextualized Reference* | The parent describes an image or experience that occurred at another time and is not in their present physical environment. The child may be prompted to connect ideas, understand an explanation, make predictions about something that has not happened, or recall something that happened in the past or the future. Decontextualized language also includes planning for the future. | P: “Remember when we went on the rollercoaster last weekend?” |
| Close Ended Questions (CEQ) | The parent or child asks a question that can be answered with one or two words or by “yes” or “no.” | P: “Do you want to read a book?”  C: “Cómo se llama?” *“What’s it called?”* |
| Open Ended Questions (OEQ) | The parent or child asks a question that requires a multiple word answer or that would require the respondent to think deeply about an answer. | P: “What was in the story?”  C: “What else, Mommy?” |

# *Note.* *Decontextualized reference was analyzed for both mother and child. All other codes were analyzed for only mothers.
